# Assessment of Handgrip Strength and Cardiac Autonomic Neuropathy Using Cardiovascular Autonomic Reflex Tests and Heart Rate Variability in Adults With Type 2 Diabetes Mellitus: A Single-Center Comparative Cross-Sectional Study

**DOI:** 10.7759/cureus.117307

**Published:** 2026-10-01

**Authors:** Sri Harini Senthilkumar, Meet Shah, Manish P Parmar, Charrumathi Dashanamurthy, Fiby George, Bhavana Gutti, Jeevankumar Murali

**Affiliations:** 1 Department of General Medicine, Government Tiruvannamalai Medical College, Tiruvannamalai, IND; 2 Department of General Medicine, B.J. Medical College, Civil Hospital, Ahmedabad, IND; 3 Department of General Medicine, Dr. N. D. Desai Faculty of Medical Science and Research, Dharmsinh Desai University, Nadiad, IND; 4 Department of General Medicine, Apollo Specialty Hospitals, Chennai, IND; 5 Department of Emergency Medicine, Marthoma Medical Mission Hospital, Ranny, IND; 6 Department of General Medicine, Bhaskar Medical College, Hyderabad, IND; 7 Department of General Medicine, Dr. Chandramma Dayananda Sagar Institute of Medical Education and Research, Bangalore, IND

**Keywords:** cardiac autonomic neuropathy, handgrip strength, heart rate variability, sarcopenia, type 2 diabetes mellitus

## Abstract

Introduction: Type 2 diabetes mellitus (T2DM) is associated with both neuromuscular impairment and diabetic autonomic neuropathy, including cardiac autonomic neuropathy (CAN). However, the relationship between handgrip strength (HGS) and cardiac autonomic dysfunction remains insufficiently characterized. This study aimed to compare HGS and cardiac autonomic parameters between adults with T2DM and healthy controls and to examine factors associated with definite CAN.

Methods: This comparative cross-sectional study included 120 adults aged 30-65 years, comprising 60 participants with T2DM and 60 age- and sex-matched controls. HGS was assessed using a calibrated dynamometer, while cardiac autonomic function was evaluated using cardiovascular autonomic reflex tests and heart rate variability analysis. Correlation analysis and multivariable logistic regression were performed.

Results: Participants with T2DM had significantly lower dominant, non-dominant, and relative HGS than controls (p<0.001). Cardiac autonomic assessment demonstrated reduced standard deviation of normal-to-normal RR intervals (SDNN) and root mean square of successive RR interval differences (RMSSD) and an increased low-frequency/high-frequency (LF/HF) ratio in the T2DM group (p<0.001 for all). Definite CAN was present in 20 (33.33%) participants with T2DM compared with four (6.67%) controls. HGS was positively associated with SDNN, RMSSD, and cardiovascular autonomic reflex indices and negatively associated with HbA1c, resting heart rate, and LF/HF ratio. Longer diabetes duration, higher HbA1c, and low HGS were independently associated with definite CAN. Low HGS showed the strongest association with definite CAN (adjusted OR=3.58, 95% CI: 1.13-11.31, p=0.030).

Conclusion: Adults with T2DM demonstrated lower HGS and greater cardiac autonomic dysfunction than healthy controls. Reduced HGS was independently associated with definite CAN after adjustment for relevant clinical factors. These findings suggest that HGS may have potential clinical value as a simple adjunctive measure when assessing autonomic involvement in adults with T2DM.

## Introduction

Type 2 diabetes mellitus (T2DM) is a public health problem that affected 200 million people globally in 1990 and more than 830 million in 2022 [1]. T2DM represents >90% of all cases of diabetes and is a consequence of the stepwise onset of insulin resistance and impaired β-cell function [2,3]. Continuous hyperglycemia gives rise to microvascular complications (retinopathy, nephropathy, neuropathy), increasing cardiovascular risk by 2-4 times [2]. In low- and middle-income countries, almost half of people with diabetes are undiagnosed, and this is the case for close to 50% of people with diabetes [4]. Multiomics-based research is beginning to show that diabetes is a disease with a multisystem nature, involving the vasculature, nerves, eyes, and kidneys [5]. Early detection is crucial to prevent the rising morbidity, mortality, and healthcare costs, which are estimated at $1054 billion in fewer than 20 years until 2045 [4].

Cardiac autonomic neuropathy (CAN) is a serious microvascular complication of diabetes and is defined by abnormalities in the functioning of the parasympathetic and sympathetic nerves that regulate cardiovascular function [6]. It occurs mainly due to chronic hyperglycemia-related inflammatory and oxidative stress affecting autonomic nerve fibers gradually [7,8]. Clinically, CAN includes resting tachycardia, exercise intolerance, orthostatic hypotension, silent myocardial ischemia, arrhythmia, and increased sudden cardiac death risk [6,8]. Early detection is critical, especially for an asymptomatic disease such as CAN, and can be obtained using cardiovascular autonomic reflex tests (CARTs) and heart rate variability (HRV) analysis for sensitive, non-invasive evaluation of autonomic nerve integrity [6-8].

Handgrip strength (HGS) is a quick and reliable indicator of overall muscle strength that is frequently used in clinical and epidemiological studies as a functional biomarker [9]. Lower HGS is clearly linked to frailty, disability, cardiovascular disease, and mortality risk and is considered a marker of underlying sarcopenia [9,10]. Muscle weakness and dysfunction are exacerbated in people with diabetes by a number of related issues, such as insulin resistance, chronic low-grade inflammation, lower levels of activity, and mitochondrial dysfunction [9].

HGS has previously been studied separately in diabetes and related cardiovascular outcomes, providing evidence for a relationship between decreased HGS and worse cardiovascular outcomes [11] and a high prevalence and mortality rate of CAN in diabetes [12]. The correlation between muscular strength and autonomic impairment, however, is still poorly defined, as there have been relatively few studies that simultaneously assessed both parameters in the same cohort of adults with T2DM [13]. A recent study by Jung et al. demonstrated that lower absolute and relative HGS were associated with the presence of CAN in patients with T2DM [14]. However, the available evidence remains limited, and differences in study populations, autonomic assessment methods, and the absence of matched non-diabetic comparison groups in some studies restrict the generalizability of these findings. Therefore, further evaluation of HGS alongside standardized cardiovascular autonomic reflex tests and HRV measures is warranted.

The present study was designed to address this gap by concurrently evaluating HGS and cardiac autonomic function in adults with T2DM and age- and sex-matched non-diabetic controls. The primary outcome was the difference in dominant HGS between the two groups. Secondary outcomes included differences in non-dominant and relative HGS, cardiovascular autonomic reflex test responses, HRV parameters, and the frequency of definite CAN. The study also examined associations between HGS, glycemic parameters, and autonomic indices and assessed whether low HGS remained independently associated with definite CAN after adjustment for relevant clinical variables. We hypothesized that adults with T2DM would demonstrate lower dominant HGS and greater cardiac autonomic dysfunction than controls and that lower HGS would be associated with definite CAN.

## Materials and methods

Study design and setting

This comparative cross-sectional study was conducted at the Department of General Medicine, Byramjee Jeejeebhoy (BJ) Medical College, Civil Hospital, Asarwa, Ahmedabad, Gujarat, India. The study was conducted over one year, from April 2025 to April 2026. The study was approved by the Institutional Ethics Committee, BJ Medical College, Civil Hospital, with an approval number: EC/Approval/24/2025/12/02/2025.

Study population

Participants with T2DM were recruited from patients attending the study center during the study period. Eligible participants were enrolled after assessment according to the predefined inclusion and exclusion criteria. The T2DM group comprised 60 adults aged 30-65 years with an established diagnosis of T2DM. The control group consisted of 60 non-diabetic adults without a history of diabetes mellitus who fulfilled the same applicable eligibility criteria.

Controls were selected to achieve comparability with the T2DM group with respect to age and sex. Participants in the two groups were therefore age- and sex-matched, with an approximately similar distribution of age and sex maintained between groups. Matching was undertaken because both age and sex may influence HGS and cardiovascular autonomic measurements. All enrolled participants subsequently underwent assessment of demographic and anthropometric characteristics, HGS, cardiovascular autonomic reflex tests, and HRV parameters.

T2DM was defined by a documented physician diagnosis and/or HbA1c ≥6.5% or fasting plasma glucose (FPG) ≥126 mg/dL. Controls had no history of diabetes and had HbA1c <5.7% and FPG <100 mg/dL at screening. Hypertension was defined by a documented diagnosis, antihypertensive treatment, or blood pressure ≥140/90 mmHg. Dyslipidemia was identified from medical history, lipid-lowering therapy, or an abnormal fasting lipid profile. Smoking status was recorded as current, former, or never smoker based on participant history. Medication history, including glucose-lowering, antihypertensive, lipid-lowering, and other drugs potentially affecting autonomic function, was documented at enrolment using the same standardized protocol applied to the T2DM group.

Inclusion criteria

The diabetes group consisted of adults (aged 30-65 years) who already had a diagnosis of T2DM for at least one year. The control group consisted of age- and sex-matched healthy individuals without diabetes mellitus. Written informed consent was used to enroll only participants.

Exclusion criteria

Participants with major neurological, cardiovascular, musculoskeletal, or other systemic conditions that could substantially affect HGS or autonomic function were excluded. Potential confounders including age, sex, BMI, hypertension, dyslipidemia, smoking status, glycemic status, and relevant medication use were documented. Measured confounders considered clinically relevant were included in the multivariable analysis evaluating factors associated with definite CAN.

Sample size calculation

The sample size was calculated for comparison of mean dominant HGS between adults with T2DM and non-diabetic controls. Based on previous literature [15], the expected mean difference in dominant HGS between the two groups was considered to be 4.50 kg, with a pooled standard deviation of 8.00 kg. Using a 95% confidence level and 80% power, the minimum required sample size was 50 participants per group, which was increased to 60 participants per group after allowing for possible non-response or incomplete autonomic recordings.

The following formula for comparison of two independent means was used:

 $n = \frac{2\left(Z_{\alpha/2}+Z_{\beta}\right)^2\sigma^2}{d^2}$

Where Zα/2 = 1.96 at 95% confidence level, Zβ = 0.84 at 80% power, σ = pooled standard deviation of 8.00 kg, and d = expected mean difference of 4.50 kg.

*n = 2 &times; (1.96 + 0.84)2 &times; 8.002 / 4.502 = 49.58*. A sample size of about 50 participants per group was required. The sample size was arrived at by rounding to 60 participants per group after adding 15.00% adjustment for possible non-response and incomplete autonomic recordings. Thus, the total sample consisted of 120 participants.

Data collection procedure

The demographic data such as age, sex, occupation, level of physical activity, and clinical history related to the study was drawn out on a proforma. The anthropometric measurements consisted of height, weight, BMI, and waist circumference. Blood pressure was obtained after a period of at least 10 minutes of rest sittings with a calibrated sphygmomanometer. The average of two readings was recorded. For the diabetic participants, duration of diabetes, treatment modality, FPG, and glycated hemoglobin were recorded. The participants were directed to refrain from caffeine, nicotine, heavy exercise, or alcohol for a minimum of 12 hours prior to testing. All autonomic function tests were performed in a quiet room after adequate rest.

Assessment of handgrip strength

HGS was measured using a Jamar Plus+ Digital Hand Dynamometer (Performance Health, USA), calibrated according to the manufacturer’s instructions before testing. Hand dominance was defined as the hand preferentially used for writing and routine daily activities. Participants were tested in a seated position with the shoulder adducted, elbow flexed at 90°, forearm in a neutral position, and wrist in slight extension. Three maximal voluntary contractions were performed with each hand, with a 60-second rest interval between successive trials, and the highest value was used for analysis. Low HGS was defined as <28 kg in men and <18 kg in women according to the Asian Working Group for Sarcopenia (AWGS) 2019 consensus criteria [16].

Assessment of cardiac autonomic function

The cardiac autonomic function was evaluated with short-term HRV and the cardiovascular autonomic reflex test. The autonomic test battery consisted of resting heart rate, heart rate response to deep breathing, Valsalva ratio, resting rate variability parameters for five minutes, the 30:15 ratio during standing, and postural fall in systolic blood pressure. Heart rate response to deep breathing was assessed during six deep breathing cycles per minute. The difference between maximum and minimum heart rate was recorded. The 30:15 ratio was calculated after standing from the supine position. The Valsalva ratio was defined as the longest RR interval following the maneuver divided by the shortest interval during the maneuver. Postural blood pressure response was determined by supine and standing systolic blood pressure. The low-frequency/high-frequency (LF/HF) ratio was assessed in the frequency domain. Decreased SDNN and RMSSD were seen as increased sympathetic modulation. Cardiac autonomic function was classified: No cardiac autonomic dysfunction: all CART results normal; early cardiac autonomic dysfunction: one abnormal CART result, definite cardiac autonomic dysfunction: two or more abnormal CARTs [17]. Cardiovascular autonomic reflex tests were interpreted using conventional Ewing criteria. An abnormal response was defined as a heart-rate variation during deep breathing ≤10 beats/min, a 30:15 ratio ≤1.00, a Valsalva ratio ≤1.10, and a fall in systolic blood pressure ≥30 mmHg following standing. These reference thresholds were applied uniformly to all participants; age-adjusted cut-offs were not used [11]. Autonomic testing was performed using a standardized protocol in a quiet, temperature-controlled room after 10 minutes of supine rest, with participants avoiding strenuous physical activity, caffeine, smoking, and alcohol before testing. Physical activity and medication use were documented, and all participants underwent testing at a consistent time of day under comparable pre-test conditions.

Short-term HRV was assessed from a five-minute resting ECG recording obtained in the supine position after at least 10 minutes of rest in a quiet, temperature-controlled room. ECG signals were acquired under standardized resting conditions, and normal-to-normal (NN) intervals were extracted for analysis. Recordings were visually inspected, and artefacts and ectopic beats were identified and excluded/corrected before HRV analysis. HRV analysis was performed using Kubios Scientific Lite™ version 4.1.2.1 (Kubios Oy, Finland). Time-domain indices included SDNN and RMSSD, while frequency-domain analysis included low-frequency (LF, 0.04-0.15 Hz) and high-frequency (HF, 0.15-0.40 Hz) components and the LF/HF ratio.

Statistical analysis

Data were entered into Microsoft Excel and analyzed using IBM SPSS Statistics for Windows, Version 24 (Released 2016; IBM Corp., Armonk, New York, United States). Continuous variables were expressed as mean ± standard deviation. Categorical variables were presented as frequencies and percentages. The Shapiro-Wilk test was used to analyze normality. An independent sample t-test was used for normally distributed continuous variables, while the Mann-Whitney U test was used for non-normally distributed variables. Categorical variables were analyzed using a chi-square test or Fisher exact test. There was a correlation between HGS and glycemic/autonomic parameters assessed using Pearson or Spearman correlation analysis. Multivariable logistic regression was performed to identify factors independently associated with definite cardiac autonomic dysfunction. Age, sex, duration of diabetes, HbA1c, and low HGS were entered into the model based on clinical relevance and univariate findings. Multicollinearity among predictors was assessed before model fitting. Adjusted odds ratios (ORs) with 95% confidence intervals (CIs) were reported, and model fit was evaluated using the Hosmer-Lemeshow test and Nagelkerke R². A p-value <0.05 was considered statistically significant.

## Results

The study included 120 participants: 60 adults with T2DM and 60 age- and sex-matched nondiabetic controls. The two groups were similar in terms of age and sex distribution (Table 1). A total of 120 participants were included, with 60 adults with T2DM and 60 age- and sex-matched controls. The two groups were comparable in age and sex distribution. Adults with T2DM showed a less favorable metabolic and cardiovascular profile, including higher waist circumference, systolic blood pressure, FPG, and HbA1c. Adequate physical activity was reported by 19 (31.67%) diabetic participants and 31 (51.67%) controls. BMI and diastolic blood pressure were comparable between the groups.

**Table 1:** Baseline demographic and clinical characteristics of study participants Values are expressed as mean ± standard deviation or n (%). An independent-samples t-test was used for continuous variables, and the chi-square test was used for categorical variables. * indicates statistical significance (p<0.05); NS: Non-significant.

Variable	Type 2 diabetes mellitus (n=60)	Controls (n=60)	Test value	p-value
Age, years	51.82 ± 8.46	50.76 ± 8.12	t=0.70	0.486^NS^
Male sex	35 (58.33%)	34 (56.67%)	chi-square=0.03	0.854^ NS^
Female sex	25 (41.67%)	26 (43.33%)	chi-square=0.03	0.854^ NS^
Body mass index, kg/m^2^	26.94 ± 3.78	25.88 ± 3.41	t=1.61	0.109^ NS^
Waist circumference, cm	92.38 ± 9.44	87.64 ± 8.92	t=2.83	0.006*
Systolic blood pressure, mmHg	128.63 ± 13.82	123.57 ± 11.94	t=2.15	0.034*
Diastolic blood pressure, mmHg	80.32 ± 8.72	78.26 ± 7.84	t=1.36	0.178^ NS^
Fasting plasma glucose, mg/dL	158.42 ± 42.76	91.34 ± 9.88	t=11.83	<0.001*
HbA1c, %	8.24 ± 1.42	5.32 ± 0.41	t=15.31	<0.001*
Adequate physical activity	19 (31.67%)	31 (51.67%)	chi-square=4.94	0.026*

Adults with T2DM demonstrated a statistically significant reduction in overall handgrip performance when compared with controls. Dominant, non-dominant, and relative HGS were all significantly lower in the diabetes group (p<0.001 for all). Sex-stratified analysis also showed significantly reduced dominant HGS among both male and female participants with diabetes (p<0.001 for both). In addition, the proportion of participants with low HGS was significantly higher among adults with T2DM (p=0.003), suggesting greater impairment of skeletal muscle strength in this group (Table 2).

**Table 2:** Comparison of handgrip strength between adults with type 2 diabetes mellitus and controls Values are expressed as mean ± standard deviation or n (%). Dominant handgrip strength represents the highest value recorded from the dominant hand. Relative handgrip strength was calculated as dominant handgrip strength divided by body weight. An independent-samples t-test and the chi-square test were applied as appropriate. * indicates statistical significance (p<0.05); NS: Non-significant.

Variable	Type 2 diabetes mellitus (n=60)	Controls (n=60)	Test value	p-value
Dominant handgrip strength, kg	24.62 ± 7.41	30.86 ± 8.26	t=4.35	<0.001*
Non-dominant handgrip strength, kg	22.83 ± 6.98	28.94 ± 7.78	t=4.53	<0.001*
Relative handgrip strength, kg/kg body weight	0.36 ± 0.12	0.47 ± 0.14	t=4.63	<0.001*
Male dominant handgrip strength, kg	28.42 ± 6.38	35.14 ± 7.20	t=4.11	<0.001*
Female dominant handgrip strength, kg	19.30 ± 4.91	25.32 ± 5.64	t=4.07	<0.001*
Low handgrip strength	26 (43.33%)	11 (18.33%)	chi-square=8.88	0.003*

Participants with T2DM showed significant impairment in cardiac autonomic function compared with controls. Resting heart rate and postural fall in systolic blood pressure were significantly higher in the diabetes group (p<0.001), indicating altered cardiovascular autonomic regulation. Parasympathetic function markers, including heart rate response to deep breathing, 30:15 ratio, Valsalva ratio, SDNN, and RMSSD, were significantly reduced among diabetic participants (p<0.001 for all). The LF/HF ratio was significantly increased in the diabetes group (p<0.001), suggesting sympathovagal imbalance with sympathetic predominance (Table 3).

**Table 3:** Comparison of cardiac autonomic function parameters between adults with type 2 diabetes mellitus and controls Values are expressed as mean ± standard deviation. BP: blood pressure; SDNN: standard deviation of normal-to-normal RR intervals; RMSSD: root mean square of successive RR interval differences; LF/HF: low-frequency/high-frequency. An independent-samples t-test was used for group comparison. * indicates statistical significance (p<0.05); NS: Non-significant.

Variable	Type 2 diabetes mellitus (n=60)	Controls (n=60)	Test value	p-value
Resting heart rate, beats/min	86.78 ± 10.42	78.10 ± 8.94	t=4.90	<0.001*
Heart rate difference during deep breathing, beats/min	11.84 ± 4.62	18.73 ± 5.42	t=7.49	<0.001*
30:15 ratio	1.05 ± 0.08	1.16 ± 0.11	t=6.27	<0.001*
Valsalva ratio	1.22 ± 0.13	1.42 ± 0.18	t=6.98	<0.001*
Postural fall in systolic BP, mmHg	13.62 ± 8.74	6.48 ± 4.86	t=5.53	<0.001*
SDNN, ms	28.94 ± 12.34	42.71 ± 15.28	t=5.43	<0.001*
RMSSD, ms	21.63 ± 10.28	36.44 ± 14.52	t=6.45	<0.001*
LF/HF ratio	2.38 ± 1.12	1.57 ± 0.74	t=4.67	<0.001*

Abnormal individual reflex tests had significantly higher prevalence in the T2DM group in all domains studied (Table 4). The distribution of abnormal cardiac autonomic function tests differed significantly between adults with T2DM and controls. Abnormal deep breathing response, abnormal 30:15 ratio, abnormal Valsalva ratio, and reduced SDNN were significantly more frequent in the diabetes group (p<0.001 for all). Orthostatic hypotension was also significantly higher among participants with T2DM (p=0.001). Overall grading of cardiac autonomic dysfunction showed a significant shift toward early and definite dysfunction in the diabetes group compared with controls (p<0.001), indicating a greater burden of cardiac autonomic impairment among adults with T2DM.

**Table 4:** Distribution of abnormal cardiac autonomic function tests and cardiac autonomic dysfunction grades Values are expressed as n (%). Cardiac autonomic dysfunction was graded based on the number of abnormal cardiovascular autonomic reflex tests. Overall comparison of autonomic dysfunction grades was performed using the chi-square test (X2); * indicates statistical significance (p<0.05). SDNN is the standard deviation of normal-to-normal RR intervals.

Variable	Type 2 diabetes mellitus (n=60)	Controls (n=60)	Test value	p-value
Abnormal deep breathing response	24 (40.00%)	5 (8.33%)	chi-square=16.33	<0.001*
Abnormal 30:15 ratio	22 (36.67%)	4 (6.67%)	chi-square=15.82	<0.001*
Abnormal Valsalva ratio	20 (33.33%)	3 (5.00%)	chi-square=15.56	<0.001*
Orthostatic hypotension	15 (25.00%)	2 (3.33%)	chi-square=11.58	0.001*
Reduced SDNN	32 (53.33%)	12 (20.00%)	chi-square=14.35	<0.001*
No cardiac autonomic dysfunction	26 (43.33%)	49 (81.67%)	chi-square=19.78	<0.001*
Early cardiac autonomic dysfunction	14 (23.33%)	7 (11.67%)	-	-
Definite cardiac autonomic dysfunction	20 (33.33%)	4 (6.67%)	-	-

Among adults with T2DM, dominant HGS showed significant negative correlations with the duration of diabetes (p=0.005), FPG (p=0.013), HbA1c (r=-0.39, p=0.002), resting heart rate (p=0.001), and LF/HF ratio (p=0.006). Significant positive correlations were observed with heart rate response to deep breathing (p=0.008), 30:15 ratio (p=0.016), Valsalva ratio (p=0.003), SDNN (p=0.001), and RMSSD (p=0.001). Body mass index was not significantly associated with dominant HGS (p=0.170) (Table 5).

**Table 5:** Correlation of dominant handgrip strength with glycemic and cardiac autonomic parameters among adults with type 2 diabetes mellitus Pearson or Spearman correlation analysis was used based on data distribution. SDNN: standard deviation of normal-to-normal RR intervals; RMSSD: root mean square of successive RR interval differences; LF/HF: low-frequency/high-frequency. * indicates statistical significance (p<0.05); NS: Non-significant.

Variable correlated with dominant handgrip strength	Correlation coefficient	p-value
Age	r=-0.30	0.020*
Duration of diabetes	r=-0.36	0.005*
Fasting plasma glucose	r=-0.32	0.013*
HbA1c	r=-0.39	0.002*
Body mass index	r=-0.18	0.170^NS^
Resting heart rate	r=-0.40	0.001*
Heart rate difference during deep breathing	r=0.34	0.008*
30:15 ratio	r=0.31	0.016*
Valsalva ratio	r=0.38	0.003*
SDNN	r=0.43	0.001*
RMSSD	r=0.41	0.001*
LF/HF ratio	r=-0.35	0.006*

Multivariable logistic regression showed that longer diabetes duration (adjusted OR=1.17, 95% CI: 1.03-1.34; p=0.019), higher HbA1c (adjusted OR=1.66, 95% CI: 1.10-2.50; p=0.016), and low HGS (adjusted OR=3.58, 95% CI: 1.13-11.31; p=0.030) were independently associated with definite cardiac autonomic dysfunction. Age (adjusted OR=1.04, 95% CI: 0.98-1.11; p=0.174) and male sex (adjusted OR=1.28, 95% CI: 0.39-4.18; p=0.682) were not significantly associated. The model showed adequate calibration (Hosmer-Lemeshow p=0.724), with a Nagelkerke R² of 0.42. No problematic multicollinearity was identified among the included covariates (Table 6).

**Table 6:** Multivariable logistic regression analysis for predictors of definite cardiac autonomic dysfunction among adults with type 2 diabetes mellitus Dependent variable: definite cardiac autonomic dysfunction. Variables were selected based on clinical relevance and significant univariate association. Adjusted odds ratio >1 indicates higher odds of definite cardiac autonomic dysfunction. Model fit: Hosmer-Lemeshow p=0.724; Nagelkerke R²=0.42. * indicates statistical significance (p<0.05); NS: Non-significant.

Predictor variable	Adjusted odds ratio	95% confidence interval	p-value
Age, per year increase	1.04	0.98-1.11	0.174^ NS^
Male sex	1.28	0.39-4.18	0.682^ NS^
Duration of diabetes, per year increase	1.17	1.03-1.34	0.019*
HbA1c, per 1% increase	1.66	1.10-2.50	0.016*
Low handgrip strength	3.58	1.13-11.31	0.030*

## Discussion

The present study shows that adults with T2DM have significantly lower HGS together with significant impairment in cardiac autonomic function when compared with sex- and age-matched healthy controls. Dominant HGS was lower in diabetic participants than in nondiabetics, while relative HGS was lower, and the prevalence of low HGS was more than twofold greater in diabetics. At the same time, the presence of autonomic dysfunction was shown by higher heart rate, lower autonomic reflex test responses, lower SDNN and RMSSD, and higher resting LF/HF ratio, and represented a definite CAN in one-third of diabetic participants. Importantly, low HGS was an independent predictor of definite CAN, indicating that muscle strength may be used as a practical surrogate marker of autonomic involvement in T2DM.

The decreased HGS we observed in our T2DM group concords with research indicating that diabetes worsens sarcopenia via insulin resistance, mitochondrial dysfunction, oxidative stress, chronic inflammation, and the development of advanced glycation end products that disrupt muscle protein synthesis and neuromuscular function [18]. The absolute and relative HGS both also decrease with age, which is a further deterioration in muscle function due to protein cross-linking in addition to increased adiposity [19,20].

Our results support prior studies showing that CAN is a significant problem with T2DM. The marked reduction in heart rate response to deep breathing (11.84 vs. 18.73 beats/min), SDNN (28.94 vs. 42.71 ms), RMSSD (21.63 vs. 36.44 ms), and elevated LF/HF ratio (2.38 vs. 1.57) reflect parasympathetic withdrawal with sympathetic predominance, consistent with HRV-based studies. Chronic hyperglycemia induces oxidative stress, endothelial dysfunction, and microvascular ischemia, which accelerates the progression of CAN and enhances cardiovascular morbidity and mortality [21-24].

Lower HGS was also positively correlated with SDNN (r=0.43), RMSSD (r=0.41), deep breathing response (r=0.34), and Valsalva ratio (r=0.38) and negatively correlated with HbA1c (r=−0.39), resting heart rate (r=−0.40), and LF/HF ratio (r=−0.35); all p-values were significant. The findings are consistent with other research finding a link between higher HGS and better resting heart rate, lower incidence of cardiovascular disease, and improved cardiopulmonary function [25-27], which provides good evidence for using HGS as a handy surrogate measure of cardiovascular and autonomic health.

The multivariable regression results confirm that diabetes duration (adjusted OR = 1.17), elevated HbA1c (adjusted OR = 1.66), and low HGS (adjusted OR = 3.58) are independent risk factors for definite CAN. Decreased HGS is associated with poor cardiovascular outcomes and mortality [28], and as time goes by, duration of the disease, poor glycemic control, and metabolic dysfunction increase autonomic injury [29,30].

Although lower HGS was associated with greater cardiac autonomic dysfunction, this association should not be interpreted as evidence that HGS can replace established autonomic assessments. HGS is a simple measure of muscle strength and may provide complementary clinical information, whereas CARTs and HRV directly evaluate different components of cardiovascular autonomic function. Therefore, HGS should be regarded as an adjunctive clinical measure rather than a substitute for CART or HRV testing.

Major strengths of this study are the age- and sex-matched design with the comparative results, the standardized HGS assessment with a dynamometer, the comprehensive assessment of autonomic function with cardiovascular autonomic reflex testing and HRV analysis, and the multivariable regression analysis to determine independent predictor values. Multiple autonomic indices and relative HGS were added to provide a full picture of the interplay between skeletal muscle physiology and cardiovascular autonomic mechanisms.

Limitations

First, the cross-sectional design does not allow for causal inference between reduced HGS and autonomic dysfunction. Second, the study was performed at a single tertiary-care center, and the sample size was not large, potentially reducing generalizability. Third, longitudinal glycemic exposure, inflammatory biomarkers, body composition, nutritional status, and objective physical activity measurements were not evaluated. Residual confounding cannot be excluded because potentially relevant factors, including BMI, hypertension, dyslipidemia, smoking status, and medication use, could not all be incorporated into the multivariable model owing to the limited number of definite CAN events. Multiple correlation analyses were performed without formal adjustment for multiple comparisons; therefore, the possibility of increased type I error was acknowledged, and borderline associations were interpreted cautiously. Moreover, future multicenter trials with cardiovascular outcome data, imaging findings, and muscle mass assessment should address whether HGS is a predictor of the progression of CAN and whether interventions to modulate muscle strength also modulate autonomic function in adults with T2DM.

## Conclusions

Adults with T2DM demonstrate significantly reduced HGS alongside impaired cardiac autonomic function compared with healthy controls. Lower HGS correlated with worse glycemic control, and autonomic indices were independently associated with definite cardiac autonomic dysfunction. HGS may represent a simple complementary clinical measure; however, prospective studies evaluating its temporal and diagnostic performance are required before its use for routine screening can be recommended.
